# Nitric Acid in Hooping Cough

**Published:** 1853-10

**Authors:** T. C. T. Arnoldi


### *On the Use of Nitric Acid in Hooping-Cough and Asthma.*

BY DR. T. C. T. ARNOLDI.

[Dr. Arnoldi recommends nitric acid as a powerful remedy in hooping-cough and asthma]:

"In hooping cough," he says, "at whatever age, whether it be a child at the breast, or a full-grown adult, I administer nitric acid in solution, as strong as lemon juice, sweetened *ad libitum.* I have given to a child of two years of age as much as one drachm and a half of concentrated nitric acid, in the above manner, per diem, and I have never known the disease to resist its use beyond three weeks. In one instance, that of a child at the breast, only seven months old, the disease disappeared within eight days. In another instance of a young lady fifteen years of age, the paroxysms were subdued within the first twenty-four hours, and the disease disappeared within ten days.—Again, in the cases of two boys about ten years of age, living at a great distance from one another, who had had the cough for several weeks, and to such a violent degree that both of them had the circumference of their eyes ecchymosed, as though they had been pommelled in pugilistic combats, the acid acted like a miracle. A medical *confrere* of mine had four of his children severely affected with the same disease in the middle of winter; and, although they had to be kept in-doors, owing to the inclemency of the weather, they were nevertheless all perfectly cured within three weeks. I might go on to cite a hundred similar instances, but these, I am satisfied, will prove sufficient to induce the profession to adopt this treatment. As regards asthma, the use of nitric acid has proved, not only in my own practice but in that of others who have adopted it, truly marvellous."

[In noticing Dr. Arnoldi's paper, the editor of the "American Journal of Medical Science" remarks, that the history of five cases of asthma, in which nitric acid was used successfully, was published in that journal by Dr. Hopkins, of Bethel, Georgia, in October, 1850.] —*Ranking's Abstract.*

